# Effects of Covering Behavior and Exposure to a Predatory Crab *Charybdis japonica* on Survival and *HSP70* Expression of Juvenile Sea Urchins *Strongylocentrotus intermedius*

**DOI:** 10.1371/journal.pone.0097840

**Published:** 2014-05-16

**Authors:** Chong Zhao, Nanjing Ji, Binglong Zhang, Ping Sun, Wenping Feng, Jing Wei, Yaqing Chang

**Affiliations:** 1 Key Laboratory of Mariculture & Stock Enhancement in North China’s Sea, Ministry of Agriculture, Dalian Ocean University, Dalian, China; 2 College of Marine Life Sciences, Ocean University of China, Qingdao, China; Laboratoire Arago, France

## Abstract

Predation is a complex process among predator, prey and environment. Juvenile sea urchins are more susceptible to predators than adults, which affects community structure. Behavior is involved in anti-predator responses by changes in the expression of anti-predator responsive genes. Here, we investigated the effects of exposure to a predatory crab *Charybdis japonica* and covering behavior on survival and *HSP70* expression of juvenile sea urchins *Strongylocentrotus intermedius*. *C. japonica* consumed large numbers of juvenile *S. intermedius* in 12 hours with a mortality of 34.17±11.43%. Covering behavior did not significantly reduce predation. Exposure to *C. japonica* did not significantly upregulate *HSP70* expression of juvenile *S. intermedius* in 12 hours. Covering behavior showed no significant regulative effect on the gene expression of *HSP70* of juvenile *S. intermedius* exposed to *C. japonica* for 12 hours. The results indicate that the anti-predator function of covering behavior is limited and that *HSP70* expression does not appear to play an important role in the anti-predator process of *S. intermedius*.

## Introduction

Sea urchins are ecologically important in structuring marine benthic communities, both as grazers and prey [1]. Predation is one of the most important biological factors affecting community structure of sea urchins [2], [3]. Compared to adults, juvenile sea urchins are more susceptible to predators than adults [4]. Thus, predation on juveniles probably plays an important role in stabilizing the large variability in recruitment and fluctuations in population density of sea urchins [4], [5], [6]. The importance of predation on juveniles in determining recruitment to urchin populations has been well reported in *Strongylocentrotus purpuratus* [7] and *Strongylocentrotus droebachiensis* [8], [9]. This highlights the ecological importance of the interaction between juvenile *Strongylocentrotus* urchins and their predators.

The sea urchin *Strongylocentrotus intermedius* is endemic to intertidal and subtidal bottoms in northern Pacific coastal waters of Hokkaido of Japan, Korea and Far East Russia [10]. It was introduced into China from Japan in 1989 for its commercial value [11]. In Japan, the mortality of juvenile *S. intermedius* transplanted to fishing grounds is high because of predation by several crab species (*Pugettia quadridens*, *Telmessus cheiragonus* and *Paralithodes brevipes*) [10]. This clearly indicates the prey-predator interaction between *S. intermedius* and crabs. The crab *Charybdis japonica*, distributed in intertidal zone and sallow water of western coast of the Pacific Ocean including China, Japan and Malaysia [12], obviously overlaps habitats with *S. intermedius* in both intertidal and subtidal zones. To our knowledge, the prey-predator relationship has never been reported between the two species, although *C. japonica* has been well documented of consuming various marine organisms, including bivalves, crustaceans and fish [13], [14], [15].

Since the risk of predation is ubiquitous, prey have evolved various behavioral strategies to minimize the impact on their survival and fitness traits [16]. Behavioral susceptibility to predator exposure has been reported in rainbow trout *Oncorhynchus mykiss* [17] and hermit crabs *Pagurus bernhardus* [18]. Covering behavior refers to sea urchins using their tube feet and spines to move objects, such as shells, stones and algae fragments, onto their dorsal surface in both shallow water [19] and the deep sea [20]. Six hypotheses have been proposed to explain the biological significance [21], including reflex action [22], ancillary feeding [23] and protection against predation [24], [25], wave surge and floating debris [26], [27], and over-exposure to light [28], [29], [30]. Among these hypotheses, protection against predation is of particular interest, because similar behaviors are involved in protective strategies of decreasing predation in terrestrial insects [31], [32]. Agatsuma (2001) [25] reported that covering behavior significantly reduced predation of juvenile sea urchins *S. intermedius* in 2 hours of exposure to the crab *P. quadridens*, but showed no significant effect after 24 and 48 hours of exposure to the predator. This clearly indicates covering behavior of *S. intermedius* has a valid but limited protective function against *P. quadridens*. However, we do not know whether covering behavior of *S. intermedius* has a similar protective function against other potential predators (for example, *C. japonica*).

An arduous challenge of behavioral biology is to investigate the physiological and molecular mechanisms of biological functions of various behaviors. Predator-induced behaviors are regulated by the expression of stress-sensitive genes [33], [34]. The family of heat shock proteins (HSP) includes a number of molecular chaperones of approximately 70kDa in size that serve indispensable roles in protein homeostasis [35]. Gene expression of *HSP*70 is involved in physiological responses to predation in damselfly larvae [36], fish [34], [37], [38] and water flea [39], although the mechanism remains largely unclear. Whether *HSP70* shows upregulation in exposure to predators remains unknown in sea urchins, although it has been well associated with thermal [40], chemical [41] and UV radiation [42] stresses. Moreover, the upregulation of *HSP70* expression was well correlated with the swimming behavior in goldfish *Carassius auratus* exposed to a predator *Lepomis macrochirus* [34]. This suggests that *HSP70* expression may be involved in the predator-induced behavior of *C. auratus*. Thus, we were motivated to investigate whether covering behavior and exposure to a predator significantly affect *HSP70* expression in *S. intermedius*.

The main purposes of the present study were to investigate 1) whether the crab *C. japonica* significantly preys on *S. intermedius*; 2) whether covering is an important behavior that significantly reduces predation of *S. intermedius* exposed to *C. japonica*; 3) whether exposure to predators and covering behavior significantly affect the gene expression of *HSP70* of *S. intermedius* exposed to *C. japonica*.

## Results

### Sea Urchins and Crabs

The body weight of sea urchins was 1.99±0.025 g (N = 240, mean ± SE), with no significant difference among all experimental groups (*P*>0.05). Carapace width, height and the length of both chelipeds of *C. japonica* showed no significant difference between the groups with and without covering material (*P*>0.05, N = 3, Table 1). However, body weight of *C. japonica* in the covering material group was significantly higher than that in the group without covering material (*P* = 0.019, N = 3, Table 1). Male and female *C. japonica* showed no significant difference in all measured traits (*P*>0.05).

**Table 1:** Sex and size traits of the crab *Charybdis japonica* (N = 3, mean ± SE).

Covering material	N	Y
Sex ratio (♂ : ♀)	2∶ 1	2∶ 1
Carapace width (cm)	7.67±0.17	8.00±0.00
Carapace height (cm)	6.00±0.00	6.00±0.00
Length of left cheliped (cm)	11.00±0.00	11.00±0.29
Length of right cheliped (cm)	11.17±0.17	11.00±0.29
Body weight (g)	98.03±3.56^a^	117.97±3.81^b^

Different letters refer to significant difference of body weight.

### Predation

**Figure 1:**
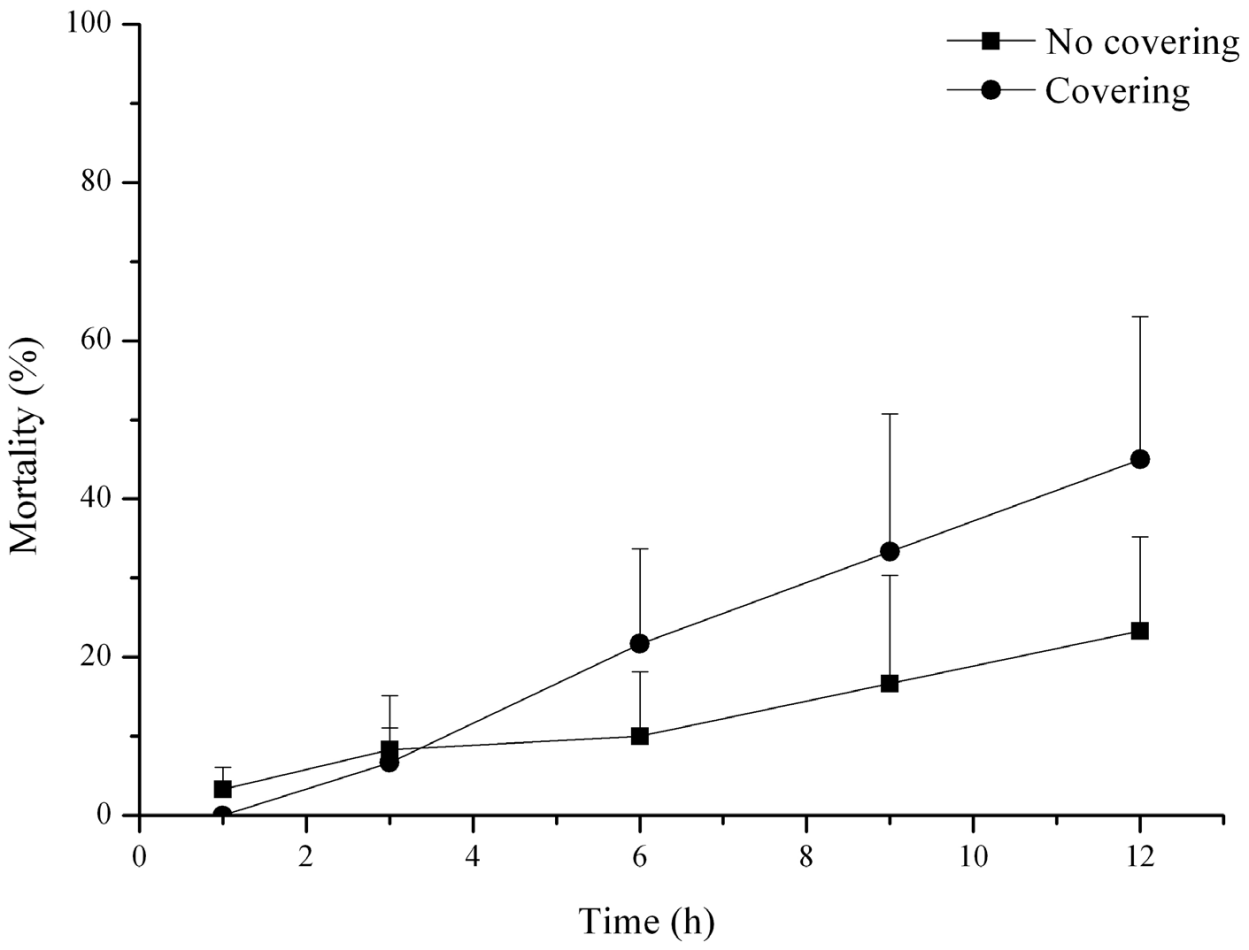
Predation on *Strongylocentrotus intermedius* with and without covering material (N = 3, mean ± SE).

There was no mortality of *S. intermedius* in the groups without predators. *C. japonica* consumed a large number of *S. intermedius* in 12 hours, with a mortality of 34.17±11.43% (*P* = 0.018, N = 3). Mortality of *S. intermedius* with covering material was obviously higher than that of individuals without covering material, although the *p* value was not significant (*P*>0.05, N = 3, Fig. 1). There was no significant interaction between covering material and predator on predation of *S. intermedius* (*P*>0.05, N = 3).

### Covering Behavior and Gene Expression of *HSP70*

**Figure 2:**
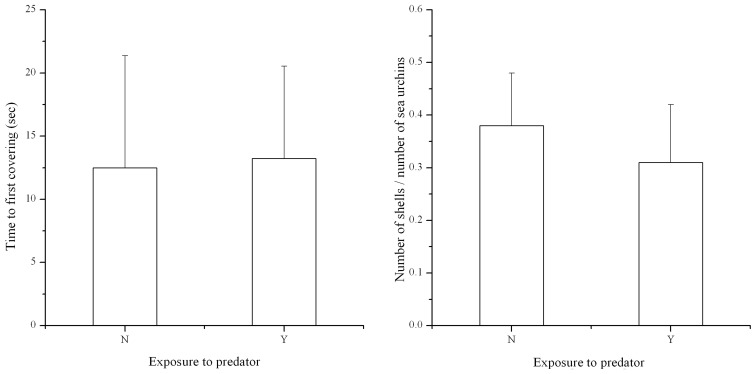
Time to first covering of *Strongylocentrotus intermedius* and number of shells covered per *S. intermedius* with or without exposure to a predator (N = 3, mean ± SE). N refers to without predator exposure, Y refers to in predator exposure.

**Figure 3:**
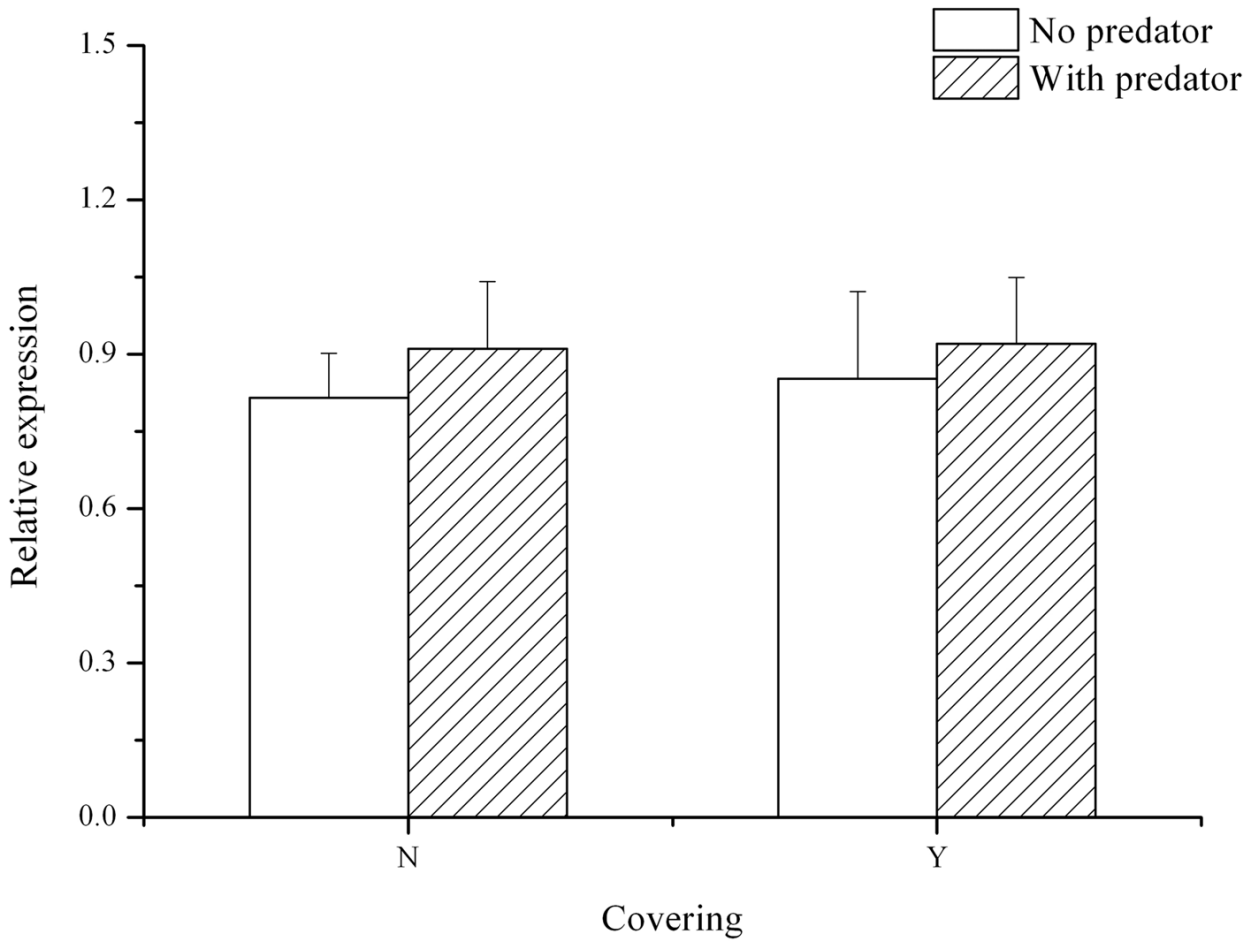
Relative expression of *HSP70* in *Strongylocentrotus intermedius* with or without the covering material and with or without exposure to a predator (N = 3, mean ± SE). N refers to without covering material, Y refers to with covering material.

Exposure to predators showed no significant effect on either time to first covering (*P*>0.05, N = 3, Fig. 2) or number of shells covered per sea urchin (*P*>0.05, N = 3, Fig. 2). Neither covering material, predator exposure nor their interaction had a significant effect on the relative expression of *HSP70* (*P*>0.05, N = 3, Fig. 3).

## Discussion

Identifying potential predators and assessing predation provide valuable ecological information about the organization of communities with implications to the conservation management of sea urchin populations [7]. To our knowledge, the relationship of prey-predator has not been documented between *S. intermedius* and *C. japonica*, although their habitats overlap in both intertidal and sublittoral zones. We observed that *C. japonica* readily consumes juvenile *S. intermedius*. This indicates that in addition to the crab *P. quadridens* [25], *C. japonica* is probably another important predator of juvenile *S. intermedius*, although further field studies should be performed to document this. Avoidance of predation by *C. japonica* would increase the survival of juvenile *S. intermedius* in regular repopulation programs for conservation and fishery in both China [11] and Japan [43].

Covering behavior appears to be a multiple-factor dependent phenomenon, although a number of hypotheses have been proposed to explain it [30]. Different from the biological significance of protection against UV light exposure, covering behavior is not well associated with benefits from protection against predation. This is because covering behavior by different sea urchin species exposed to different predators showed different effects [24], [25]. In the present study, we found that availability of covering material failed to significantly increase the survival of juvenile *S. intermedius* after 12 hours of exposure to *C. japonica*. Our result is consistent with the finding of Agatsuma (2001) [25] that covering behavior did not have a significant protective effect on survival of juvenile *S. intermedius* after 24 and 48 hours of exposure to *P. quadridens*, but significantly reduced predation of juvenile *S. intermedius* after 2 hours of exposure to *P. quadridens*. It should be noted the *C. japonica* used in the present study were much larger than the *P. quadridens* used by Agatsuma (2001) [25]. These results suggest that covering behavior is an effective protective strategy against predators in short-term exposure to some special predators. This can be partly supported by the finding of Amsler et al. (1999) [24] that the survival rate of covered sea urchins *Sterechinus neumayeri* was significantly higher than uncovered individuals exposed to sea anemone *Isotealia antarctica*. There was a trend of higher mortality of sea urchins with covering material than that of individuals without covering material, although the *p* value was not significant. A possible explanation is that body weight of *C. japonica* in the covering group was significantly higher than those in the group without covering material. Another limitation of the present study is that only three crabs of both sexes (two males and one female) were used in each experimental group. Male and female *C. japonica* in the present study showed no significant difference in all measured traits, although sexual difference in claws was documented in another crab species *Pachygrapsus crassipes* [7]. In addition, the ratio of males:females was constant and would not affect overall predation. However, the limited number of *C. japonica* of both sexes probably reduces the robustness of the statistical analysis. Together with previous studies, the present study indicates that crabs probably play an important role in the complex predator - prey interaction of sea urchins and that the protective effect of covering material seems much weaker than habitat refuges [7].

We expected exposure to predators might significantly increase covering behavior of juvenile *S. intermedius*. However, we found that predation did not significantly increase covering behavior, either in time to first covering or number of shells covered per sea urchin. This result is consistent with the study of Dumont et al (2007) [30], who found that predator stimulus showed no significant effect on covering behavior of the sea urchin *S. droebachiensis*. Therefore, covering behavior does not appear to play an important role in the anti-predator response of sea urchins in exposure of starved and voracious predators.

Upregulation of *HSP70* has been linked to antipredator responses in both invertebrates [36], [39] and vertebrates [34], [37], [38]. In the present study, although predation was high, neither exposure to predators, covering behavior nor their interaction showed significant difference in relative expression of *HSP70*. This result is consistent with Sørensen et al. (2011) [44], who reported that 96 hours of predator exposure did not induce significant response of *HSP70* expression in tadpoles of the frog *Rana temporaria*. There are two possible explanations. One possibility is that an inducible response of *HSP70* expression in juvenile *S. intermedius* would be missed after 12 hours of exposure to *C. japonica* because the quickness of *HSP70* expression response to the exposure of predators is species dependent [44]. For example, *HSP70* expression in goldfish (*C. auratus*) showed a significant rapid upregulation in 6 hours exposure of a predator fish [37]. However, Slos & Stoks (2008) [36] reported a slow significant response in *HSP70* expression of 5 days in the damselfly *Enallagma cyathigerum* under a predation risk. Another possibility is other anti-predator sensitive genes besides *HSP70* in juvenile *S. intermedius* responded to the 12h exposure of *C. japonica*. Further studies are required to investigate whether *HSP70* expression is related to predation in other urchin species under various environmental and predation conditions.

In conclusion, the crab *C. japonica* preyed on juvenile *S. intermedius*. Covering behavior did not significantly reduce the mortality of juvenile *S. intermedius* in 12 hours of exposure to *C. japonica*. Exposure to *C. japonica* did not significantly upregulate *HSP70* expression in 12 hours. Covering behavior showed no significant regulative effect on the gene expression of *HSP70* of juvenile *S. intermedius* exposed to *C. japonica* for 12 hours. The present results indicate that the anti-predator function of covering behavior is limited in the exposure to starved and voracious predators and that *HSP70* appears to not play an important role in the anti-predator process in juvenile *S. intermedius*. Compared to the experimental data in insects [31], [32], [36], the present study clearly suggests that anti-predator strategies are highly species dependent.

## Materials and Methods

### Sea Urchins and Crabs

Two hundred and forty juvenile *S. intermedius* approximately 10–20 mm in test diameter, from a batch produced in October, 2012, were transported from the Dalian Haibao Fisheries Company to the Key Laboratory of Mariculture & Stock Enhancement in the North China’s Sea, Ministry of Agriculture at Dalian Ocean University on July 15, 2013. They were maintained at 22–24°C and under natural sunlight in 200L tanks and fed kelp *Laminaria japonica* ad libitum until the experiment began on August 6, 2013. The body weight of all sea urchins was measured before the experiment. To avoid potential harms to juvenile urchins, we did not precisely measure their test diameter and height.

Six crabs *C. japonica* were locally caught at Heishijiao, Dalian (38°52′ N, 121°34′ E), where no specific permission for this activity is required. They were then separately maintained under the same culture condition of *S. intermedius* and fed clams (*Ruditapes philippinarum*) in the laboratory to ensure that they were capable of feeding and not moulting. They were starved for one week before the experiment started. The size and sex of the six crabs were recorded before the experiment. The field studies did not involve endangered or protected species.

### Experimental Design

The present study included three experiments. Experiments were carried out with sea urchins at 23.7–26.0°C, 31 ‰ salinity, and pH 8.12 and under natural sunlight. The experiment began on August 6, 2013. First, we investigated the mortality of *S. intermedius* exposed to *C. japonica* with and without covering material (presence of covering material and predator as the two factors). *C. japonica* were individually put into six experimental tanks (70L) with or without covering material. Two males and one female *C. japonica* were put into the experimental groups with and without covering material to ensure a constant ratio of males:females in each group. Six similar tanks without predators were set up as controls. According to our previous study [45], twenty shells of the small bivalve *Patinopecten yessoensis* (shell length: 20.31±0.99 mm, shell height: 21.54±1.10 mm, shell weight: 0.28±0.06 g) were used as potential covering material and distributed evenly on the bottoms of the experimental tanks. Twenty sea urchins were gently and evenly distributed onto the bottom of each experimental tank. Mortalities of *S. intermedius* in all experimental groups were recorded after 1h, 3h, 6h, 9h and 12h. The second experiment was the behavioural tests with and without predators (presence of predator as the factor). We recorded the time to first covering (behavioral action time) and the number of shells covered per sea urchin 12 hours after the beginning of the experiment (behavioral ability) for each of the six experimental tanks. The third experiment was the relative expression of *HSP70* by the sea urchins with and without exposure to the predator and with and without the presence of covering material (presence of covering material and predator as the two factors). Twelve hours after the beginning of the experiment, coelomic fluid collected from each sea urchin using a 1mL syringe was centrifuged at 3000×rpm for 2 minutes (4°C) to harvest coelomocytes. All the samples were immediately frozen in liquid nitrogen and then stored at −80°C until used.

### Gene Expression of *HSP70*

Total RNA was extracted with the total RNA kit (TIANGEN, China) following the manufacturer instructions. First strand cDNA was synthesized using the PrimeScrip RT reagent Kit (TaKaRa, Japan) according to the manufacturer instructions.

To obtain the *HSP70* expression profile, cDNA samples were analyzed with quantitative RT-PCR. Gene specific primers 5′-ACACTCATCTCGGAGGAG-3′ (*HSP70*-F) and 5′-CTTTCTTATGCTTTCGCTTGA-3′ (*HSP70*-R) were designed for the HSP70 fragments by Primer Premier 5.0 software. The sea urchin 18S ribosomal RNA was used as the reference gene. It was amplified using gene-specific primers of 5′-GTTCGAAGGCGATCAGATAC-3′ (18S-F) and 5′-CTGTCAATCCTCACTGTGTC-3′ (18S-R) [46]. The qRT-PCR was carried out in a 20 µL volume including 10 µL of 2×SYBR Green Master mix (TaKaRa, Japan), 0.4 µL ROX Reference Dye II, 1 µL of 1∶5 diluted cDNA, 0.4 µM of each primer and 7 µL ddH_2_O using the Applied Biosystem 7500 Real-time system (Applied Biosystem, USA). The PCR condition was set as: 95°C for 30 s, followed by 40 cycles of 95°C for 5 s and 60°C for 32 s. Melting curve analysis of amplification products was performed at the end of each PCR reaction to confirm amplification specificity. The comparative Ct method was used to calculate the relative expression levels of the *HSP70* [47].

### Statistical Analysis

Mortalities of *S. intermedius* in the exposure to *C. japonica* with and without covering material were analyzed using two-way repeated measured ANOVA (presence of covering material and predator as the two factors with repeated measures on time). Time to first covering and the number of shells covered per sea urchin at 12h were analyzed by one-way ANOVA (presence of predator as the factor). Relative expression of *HSP* in *S. intermedius* was analyzed by two-way ANOVA (presence of covering material and predator as the two factors). All analyses were performed with SPSS 13.0 statistical software. A probability level of *P*<0.05 was considered statistically significant.
